# Disentangling dyskinesia from parkinsonism in motor structures of patients with schizophrenia

**DOI:** 10.1093/braincomms/fcac190

**Published:** 2022-07-23

**Authors:** Katrin Sakreida, Wei-Hua Chiu, Juergen Dukart, Simon B Eickhoff, Thomas Frodl, Christian Gaser, Michael Landgrebe, Berthold Langguth, Daniela Mirlach, Ioana-Sabina Rautu, Markus Wittmann, Timm B Poeppl

**Affiliations:** Department of Psychiatry, Psychotherapy and Psychosomatics, Faculty of Medicine, RWTH Aachen University, Aachen 52074, Germany; Department of Neurology, Grossman School of Medicine, New York University, New York, NY 10017, USA; Institute of Neuroscience and Medicine (INM-7), Research Centre Jülich, Jülich 52425, Germany; Institute of Systems Neuroscience, Heinrich Heine University Düsseldorf, Düsseldorf 40225, Germany; Institute of Neuroscience and Medicine (INM-7), Research Centre Jülich, Jülich 52425, Germany; Institute of Systems Neuroscience, Heinrich Heine University Düsseldorf, Düsseldorf 40225, Germany; Department of Psychiatry, Psychotherapy and Psychosomatics, Faculty of Medicine, RWTH Aachen University, Aachen 52074, Germany; Department of Neurology, Jena University Hospital, Jena 07747, Germany; Department of Psychiatry and Psychotherapy, Jena University Hospital, Jena 07743, Germany; Department of Psychiatry, Psychotherapy and Psychosomatics, kbo-Lech-Mangfall-Klinik Agatharied, Hausham 83734, Germany; Department of Psychiatry and Psychotherapy, University of Regensburg, Regensburg 93053, Germany; Department of Psychiatry and Psychotherapy, University of Regensburg, Regensburg 93053, Germany; Unité de Recherche en Neurosciences Cognitives (UNESCOG), Center for Research in Cognition and Neurosciences (CRCN), Université Libre de Bruxelles (ULB), Brussels 1050, Belgium; Department of Psychiatry, Psychosomatics and Psychotherapy, District Hospital Wöllershof, Störnstein 92721, Germany; Department of Psychiatry, Psychotherapy and Psychosomatics, Faculty of Medicine, RWTH Aachen University, Aachen 52074, Germany; Department of Psychiatry and Psychotherapy, University of Regensburg, Regensburg 93053, Germany

**Keywords:** motor symptoms, schizophrenia, grey matter volume, magnetic resonance imaging, neurotransmitters

## Abstract

Patients with schizophrenia frequently suffer from motor abnormalities, but underlying alterations in neuroarchitecture remain unclear. Here, we aimed to disentangle dyskinesia from parkinsonism in motor structures of patients with schizophrenia and to assess associated molecular architecture. We measured grey matter of motor regions and correlated volumetric estimates with dyskinesia and parkinsonism severity. Associations with molecular architecture were identified by cross-modal spatial correlations between ensuing maps of abnormality-related volume alterations and neurotransmitter maps from healthy populations. Both phenomena were linked to (specific) striatal and basal forebrain reductions as well as to D_1_ receptor density. Dyskinesia also manifested in cerebellar decrease, while parkinsonism was associated with less motor cortex volume. The parkinsonism-related brain pattern was additionally associated with 5-HT_1A/2A_ and µ-opioid receptors distribution. Findings suggest the need to develop psychopharmacological compounds that display not only selectivity for receptor subtypes but also anatomical selectivity for alleviating dyskinesia without worsening parkinsonism and vice versa.

## Teaser

Sakreida *et al*. identify common as well as distinct structural brain changes and associated molecular mechanisms linked to dyskinesia and parkinsonism in schizophrenia. Motor abnormality-specific effects suggest the need for psychopharmacological compounds with selectivity for not only receptor subtypes but also anatomy.

## Graphical Abstract

**Graphical abstract:**
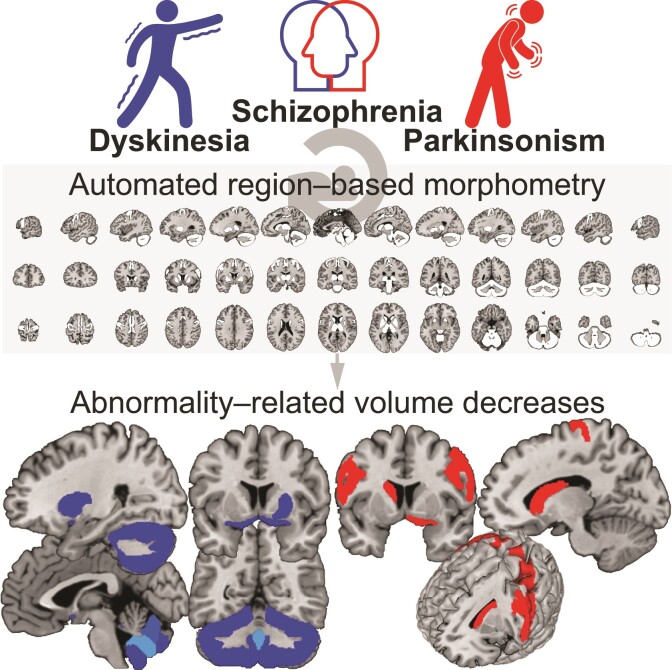

## Introduction

Patients with schizophrenia frequently suffer from motor abnormalities including hyper- and hypokinetic syndromes such as dyskinesia and parkinsonism. Both dyskinesia and parkinsonism represent common side effects of antipsychotic treatment in at least 25% of patients.^1–3^ They also occur in medication-naïve patients with comparable prevalence rates.^4,5^ Moreover, significantly increased prevalence rates even in unaffected first-degree relatives of patients with schizophrenia suggest that abnormalities in neural motor pathways are not only associated with schizophrenia itself but also subject to genetic susceptibility.^6^

The first magnetic resonance imaging (MRI) study in medicated schizophrenia patients with and without dyskinesia indicated decreased volumes of the caudate nuclei but not of other basal ganglia structures.^7^ However, subsequent studies investigating chronic patients with schizophrenia could not replicate this finding and suggested opposite effects in other basal ganglia regions, i.e. increased size of the lentiform nucleus.^8,9^ Similarly, reported striatal abnormalities associated with parkinsonism were initially not replicated.^8,10^ A review of the literature concluded that brain alterations related to dyskinesia and parkinsonism in schizophrenia still remain inconclusive.^11^ However, previous literature suggests that (differential) alterations are not limited to subcortical motor structures (i.e. basal ganglia and cerebellum) but also concern cortical motor structures.^1^ Despite co-occurrence of both motor phenomena in the course of the schizophrenic disease process,^5^ dyskinesia and parkinsonism were mostly investigated in different samples and with varied neuroanatomical focus, limiting interpretation with respect to the specificity of the findings. Animal models suggest that dyskinetic and parkinsonian states are based on diametric pathophysiology, which is mediated by imbalance of the neurotransmitter dopamine in striatal pathways.^12^ However, it remains largely unknown how putative neuroanatomical alterations associated with dyskinesia and parkinsonism relate to neurotransmitter systems.

We hypothesized that dyskinesia and parkinsonism in schizophrenia are associated with specific alterations in both cortical and subcortical motor structures. Moreover, we conjectured that the corresponding patterns of alterations relate to the dopaminergic system but also to specific distribution of other receptors. Here, we used automated region-based morphometry of anatomical MRI data to disentangle dyskinesia from parkinsonism in motor structures of patients with schizophrenia. To assess their molecular underpinnings, the identified patterns of grey matter volume alterations were spatially correlated with density maps of 11 receptors/transporters covering various neurotransmitter systems as derived from molecular imaging in healthy populations.

## Materials and methods

### Participants, clinical assessments and imaging

We included 35 patients (13 females, mean age = 39.3 ± 13.7 years, range 18–64 years) who met the ICD-10 criteria for schizophrenia. Severity of motor symptoms was rated by trained psychiatrists on two established clinical scales. We employed the Abnormal Involuntary Movement Scale (AIMS)^13^ to quantify dyskinesia and the Simpson–Angus Scale (SAS)^14^ to assess parkinsonism. Psychopathological symptoms were measured using the Brief Psychiatric Rating Scale (BPRS).^15^ All patients were on antipsychotic medication. Seven different first-generation and eight different second-generation antipsychotics were administered in the whole sample. Four patients were exclusively medicated with first-generation antipsychotics, 25 patients exclusively received second-generation antipsychotics, and 6 patients were on antipsychotics of both generations. T1-weighted anatomical images were acquired on two 1.5 Tesla Siemens MRI scanners and preprocessed using standard pipelines (see Supplementary Methods). Patients from both scanners did not differ with respect to sex and age nor regarding all collected clinical variables including age at disease manifestation, disease duration, times of being inpatient, concomitant antipsychotic dose as well as BPRS, AIMS and SAS scores (all *P*-values ≥ 0.093). All patients provided written informed consent. All study procedures were in accordance with the Declaration of Helsinki and had been approved by the ethics committee of the University of Regensburg.

### Region-based morphometry

Individual MRI data were preprocessed using the Computational Anatomy Toolbox (CAT; Version 12.6) as an extension to the Statistical Parametric Mapping (SPM) software (Version 12; SPM12; Wellcome Trust Centre for Neuroimaging, London, UK). Preprocessing includes the standard options of segmentation and normalization to the Montreal Neurological Institute template as implemented in SPM12. After quality check for sample homogeneity, data were smoothed using a Gaussian filter with a full-width at half-maximum smoothing kernel of 8 mm.

We defined 26 individual regions of interest (ROI) in the motor system including motor cortices, basal forebrain, basal ganglia, thalamus, cerebellum and brainstem (see Supplementary Table 1 for an exhaustive list). Maximum probability tissue labels derived from the Neuromorphometrics atlas were used to estimate the mean value of local grey matter volume inside the defined ROI. The anatomical atlas, which is defined in template space, was transformed to native subject space using the inverse non-linear deformations needed to spatially normalize images to template space. Total intracranial volume was estimated for each patient and used as a nuisance variable in the statistical analyses. There was no difference regarding relevant variables such as sex and age as well as all collected clinical variables between patients from both scanners (see Supplementary Methods). Therefore, sample was not modelled as covariate.

### Neurotransmitter mapping

We used the JuSpace toolbox^16^ for cross-modal spatial correlation analyses of MRI data with positron emission tomography–derived estimates covering various receptor systems including dopaminergic (dopamine D_1_ and D_2_; dopamine transporter: DAT, dopamine synthesis capacity: FDOPA), serotonergic (serotonin 5-hydroxytryptamine receptor subtypes 1a, 1b and 2a: 5-HT_1A_, 5-HT_1B_, 5-HT_2A_; serotonin transporter: SERT), noradrenergic (noradrenaline transporter: NAT), μ-opioid and gamma-aminobutric acid (GABA)ergic (GABA_A_) neurotransmission as obtained from healthy volunteer studies. Analyses were based on unthresholded grey matter brain maps modelling the correlation between volume reduction and severity of dyskinesias and parkinsonism (i.e. AIMS and SAS scores).

### Statistical analysis

We used IBM® SPSS® Statistics for Mac (Version 25.0) for the statistical analyses. Partial correlations between extracted grey matter volumes in the motor ROI and severity of dyskinesia (AIMS sum score of 10 items) and parkinsonism (mean of SAS single scores) were calculated and deemed significant at *P* < 0.05, corrected for 26 (multiple) comparisons using the false discovery rate (FDR).^17^

A Spearman correlation analysis was performed for each of the 11 receptors/transporters provided by the JuSpace toolbox correlating its spatial distribution with local grey matter volumes on the group level using the Neuromorphometrics atlas. We used the default option accounting for spatial autocorrelation. Spearman ϱ correlation coefficients were Fisher’s *z*-transformed. Receptors showing a significant [*P* < 0.01, FDR corrected for 11 (multiple) comparisons] association with grey matter volumes were entered into a multiple linear regression analysis to disentangle their specific associations.

## Results

### Relationship of dyskinesia and parkinsonism with clinical characteristics

Age at disease manifestation, concomitant antipsychotic dose and current psychopathology did not significantly correlate with severity of dyskinesia (AIMS scores, mean = 4.6 ± 6.2, range 0–24) or of parkinsonism (SAS scores, mean= 0.5 ± 0.4, range 0–1.8; −0.138 ≤ all *r* ≤ 0.300 and all *P*-values ≥ 0.089; see Supplementary Table 2). That is, severity of both motor phenomena was not linked to early disease manifestation, high concomitant antipsychotic dose or severity of psychopathological symptoms. However, both disease duration and times of being inpatient were significantly positively correlated with dyskinesia scores (*r*_s_ = 0.712, *P* < 0.001; *r*_s_ = 0.538, *P* = 0.002), but not with severity of parkinsonism (*r*_s_ ≤ 0.284, *P* ≥ 0.115). This relationship thus indicates that chronification of disease may be a significant risk factor for the development of dyskinesia.

### Altered grey matter morphology related to dyskinesia and parkinsonism

**Figure 1:**
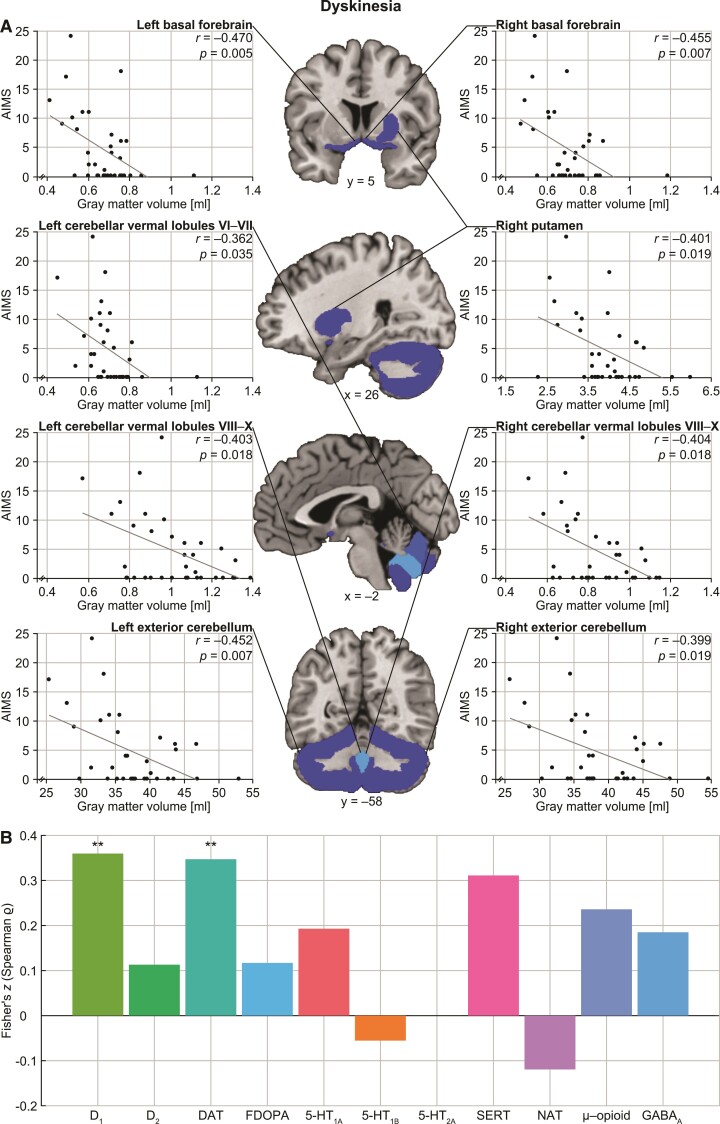
**Grey matter decrease associated with dyskinesia and relationship to molecular architecture.** (**A**) Our partial correlation analyses showed significant negative relationships between grey matter volumes of bilateral basal forebrain, right putamen as well as several portions of the cerebellum with AIMS scores. (**B**) The corresponding brain map of dyskinesia-related changes was positively associated with availability of the dopamine D_1_ receptor as well as dopamine transporter capacity, as calculated by Spearman correlation analysis. **Significant at *P* < 0.01, FDR corrected.

**Figure 2:**
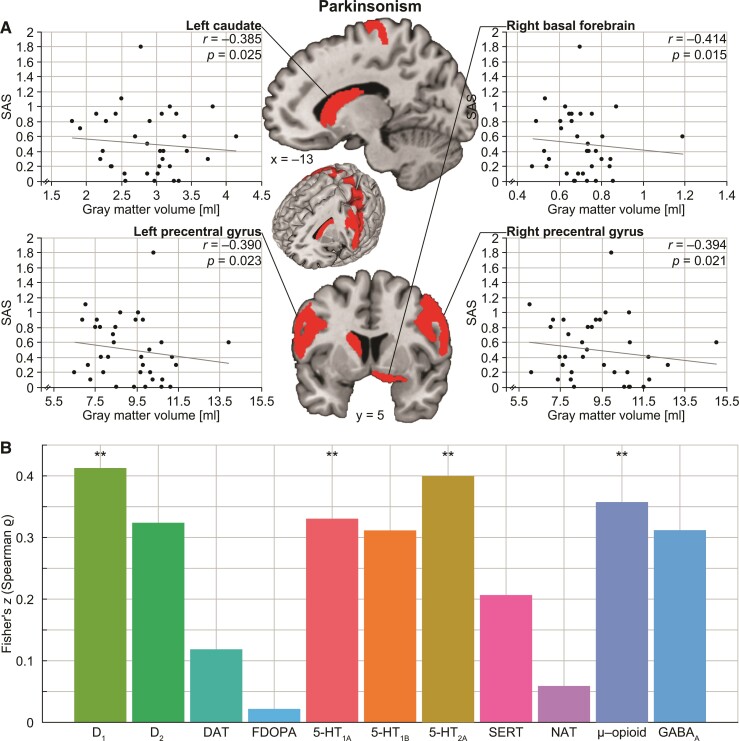
**Grey matter decrease associated with parkinsonism and relationship to molecular architecture.** (**A**) Our partial correlation analyses showed significant negative correlations between grey matter volumes of bilateral precentral gyrus, left caudate as well as right basal forebrain with SAS scores. (**B**) The corresponding brain map of parkinsonism-related changes was positively associated with availability of the dopaminergic, serotonergic and opioid receptors, as calculated by Spearman correlation analysis. **Significant at *P* < 0.01, FDR corrected.

We found significant negative correlations of grey matter volumes with severity of dyskinesia in bilateral basal forebrain, right putamen and several portions of cerebellum [all *r* ≤ −0.362, all *P*-values (two-tailed) ≤ 0.035; Fig. 1A). Using the AIMS sum score of Items 1–7 instead of all 10 items did not significantly change the results. Furthermore, our analyses revealed significantly decreased grey matter volumes associated with severity of parkinsonism in bilateral precentral gyrus, left caudate nucleus and right basal forebrain (all *r* ≤ –0.385, all *P*-values (two-tailed) ≤ 0.025; Fig. 2A). That is, both dyskinesia and parkinsonism were linked to basal forebrain pathology but affected the striatum differently, the first related to alteration of the right putamen and the latter to changes in the left caudate. Moreover, while dyskinesia additionally manifested in cerebellar decrease, parkinsonism showed effects in cortical motor structures.

### Relationship to molecular architecture

Correlation analyses with molecular imaging–derived neurotransmitter maps identified significant relationships of the brain map of dyskinesia-related grey matter alterations with dopaminergic neurotransmitter systems [(receptor/transporter, *r*_s_, *P*-value) D_1_: *r*_s_ = 0.345, *P* = 0.005; DAT: *r*_s_ = 0.334, *P* = 0.006; Fig. 1B]. The map of parkinsonism-related grey matter changes was significantly correlated with dopaminergic, serotonergic and opioid neurotransmitter systems (D_1_: *r*_s_ = 0.391, *P* = 0.001; 5-HT_1A_: *r*_s_ = 0.319, *P* = 0.009; 5-HT_2A_: *r*_s_ = 0.380, *P* = 0.002; μ-opioid: *r*_s_ = 0.343, *P* = 0.005; Fig. 2B). That is, the higher the availability of the respective receptors as derived from healthy volunteer studies, the higher the dyskinesia- and parkinsonism-related grey matter loss.

When testing for specificity of the above findings by controlling for each other impact in dyskinesia- and parkinsonism-related neurotransmitter systems using multiple linear regression, we found that among both significant associations between dyskinesia-related grey matter alterations and dopaminergic neurotransmitter systems, the dopamine transporter survived at a marginal significance level (*P* = 0.070). Among the parkinsonism-related associations, the 5-HT_2A_ (*P* = 0.012) and the μ-opioid receptor (*P* = 0.004) remained significant.

## Discussion

We found a reduction of grey matter volumes in the right basal forebrain linked to higher severity of both motor phenomena but differentially altered striatal volume, i.e. a dyskinesia-related effect on the right putamen and a parkinsonism-related effect on the left caudate. In addition, higher dyskinesia severity was associated with cerebellar volume decrease, whereas parkinsonism-related volume decrease was observed in the motor cortex. The brain pattern of both dyskinesia- and parkinsonism-related alterations was linked to the dopaminergic neurotransmitter system. Parkinsonism-related changes were additionally related to serotonergic and opioid systems.

Our results align previous, partly inconsistent findings regarding involvement of basal ganglia and particularly striatum in dyskinesia and parkinsonism in patients with schizophrenia.^7–10,18^ The observed striatal pathology must not necessarily be driven by antipsychotic-medication effects but might also reflect general schizophrenia pathophysiology.^19^ The observed alterations of basal ganglia and related forebrain structures linked to both dyskinesia and parkinsonism are in line with their involvement in the pathophysiology of hyper- and hypokinetic disorders such as chorea/ballism and Parkinson’s disease.^20,21^ The identified spatial relationship between the maps of structural motor-system alterations and dopaminergic receptor/transporter distribution corroborates the hypothesis that decreased dopamine concentrations and dopamine receptor hypersensitivity in the nigrostriatal pathway are crucial pathophysiological mechanisms of parkinsonism and dyskinesia, respectively.^3^

In a similar vein, our finding of decreased cerebellar volume associated with dyskinesia severity straightens previous inconsistent results regarding cerebellar abnormalities in schizophrenia patients with dyskinesia.^18,22^ In addition, it matches well with the model proposing a key role of the cerebellum in the generation of levodopa-induced dyskinesia in non-schizophrenic patients.^23^ Our analyses also complement previous evidence of parkinsonism-related grey matter decrease in the motor cortex of patients with schizophrenia^24^ by showing that the amount of loss is not merely categorical but correlated with parkinsonism severity.

The parkinsonism-related pattern of motor system alterations in patients with schizophrenia was also associated with the serotonergic neurotransmitter system. Schizophreniform symptoms in Parkinson’s disease have been conceptualized as an imbalance between dopaminergic and serotonergic neurotransmission,^25^ given that serotonin 5-HT receptors indirectly modulate motor activity by regulating release of dopamine in the nigrostriatal pathway.^26,27^ The phenomenon-specific, anatomy-neurotransmitter associations observed in our study further support the notion that, in addition to selectivity for 5-HT_1A_ and 5-HT_2A_ receptor subtypes, it will be necessary to develop compounds that display anatomical selectivity in order to alleviate dyskinesia without worsening parkinsonism and vice versa.^28,29^ That our analyses linked parkinsonism-related alterations to the opioid system is in line with known enhancement of opioid transmission in the basal ganglia in Parkinson’s disease.^30^ In this context, this finding may further explain why κ- and µ-opioid receptor agonists seem to lead to improvement of parkinsonism.^31,32^

In this context, it has to be kept in mind that these findings are based on correlation analyses between neuroanatomical changes in patients and neurotransmitter maps as derived from healthy populations. It might be assumed, however, that patients with chronic schizophrenia would exhibit relevant alterations of receptor availability in these brain areas. In other words, the correlations may not hold true for the association with severity of motor abnormality in psychosis. Nevertheless, our findings provide a sound basis for further investigations of these associations, using direct measures of receptor distribution and transporter availability in patients with chronic schizophrenia. A limitation of our study is that it cannot definitely exclude that catatoniform symptoms, which overlap with parkinsonism, influence the results. Moreover, although the SAS is commonly used in antipsychotic drug trials, it has the drawback of overemphasizing the assessment of rigor. Hence, our findings regarding parkinsonism might represent effects of rigor rather than of tremor or deficient postural control. In addition, it has to be considered that the result of lacking direct association between severity of motor abnormalities and antipsychotic medication is based on concomitant antipsychotic dosage and not on estimation of total antipsychotic exposure during the lifespan. Future analogue studies should re-examine this association, if the corresponding data are available. Such future studies should also increase the sample size and thereby statistical power to assure that the observed effects generalize to a larger, even more heterogeneous cohort of patients with schizophrenia.

Taken together, our data suggest that both dyskinesia and parkinsonism in schizophrenia are linked to (specific) basal ganglia and forebrain pathology and the dopaminergic system, but differ in involvement of cerebellum and motor cortex as well as of serotonergic and opioid neurotransmission. These attributes may guide the development of drugs that can balance more specifically antipsychotic and motor (side) effects.

## Supplementary Material

fcac190_Supplementary_Data

## Data Availability

The data that support the findings of this study are available from the corresponding author, upon reasonable request.

## Abbreviations

AIMS =: Abnormal Involuntary Movement Scale
BPRS =: Brief Psychiatric Rating Scale
FDR =: false discovery rate
ROI =: regions of interest
SAS =: Simpson–Angus Scale
SPM =: Statistical Parametric Mapping
