# Supplementary material for: Disentangling dyskinesia from parkinsonism in motor structures of patients with schizophrenia
Source: Brain Commun. 2022 Jul 23;4(4):fcac190. doi: 10.1093/braincomms/fcac190 (PMC9337227; doi:10.1093/braincomms/fcac190)
Supplement: fcac190_Supplementary_Data [file fcac190_supplementary_data.pdf]

Supplementary online-only material

Supplementary methods

Three-dimensional anatomical data were acquired on two 1.5 Tesla Siemens (Erlangen, Germany) magnetic resonance imaging scanners using T1-weighted sequences with the following parameters: MAGNETOM Sonata: field of view = 256 × 256, 176 sagittal slices, slice thickness = 1 mm, voxel dimension = 0.977 mm × 0.977 mm × 1 mm, repetition time = 1,880 ms, echo time = 3.93 ms, flip angle = 15°; MAGNETOM Aera: field of view = 256 × 256, 160 sagittal slices, slice thickness = 1 mm, voxel dimension = 1 mm × 1 mm × 1 mm, repetition time = 2,060 ms, echo time = 5.99 ms, flip angle = 15°.

Supplementary methods table

Supplementary Table 1. Regions of interest (ROIs) in the brain's motor system.

ROI name	ROI abbreviation
Left precentral gyrus	lPrcGy
Right precentral gyrus	rPrcGy
Left medial (segment of) precentral gyrus	lMedPrcGy
Right medial (segment of) precentral gyrus	rMedPrcGy
Left cerebrum and motor (supplementary motor area)	lCbr+Mot
Right cerebrum and motor (supplementary motor area)	rCbr+Mot
Left basal cerebrum and forebrain	lBasCbr+FobBr
Right basal cerebrum and forebrain	rBasCbr+FobBr
Left Caudate	lCau
Right Caudate	rCau
Left Pallidum	lPal
Right Pallidum	rPal
Left Putamen	lPut
Right Putamen	rPut
Left thalamus proper	lThaPro
Right thalamus proper	rThaPro
Left exterior cerebellum	lExtCbe
Right exterior cerebellum	rExtCbe
Left cerebellar vermal lobules I-V	lCbeLoCbe1-5
Right cerebellar vermal lobules I-V	rCbeLoCbe1-5
Left cerebellar vermal lobules VI-VII	lCbeLoCbe6-7
Right cerebellar vermal lobules VI-VII	rCbeLoCbe6-7
Left cerebellar vermal lobules VIII-X	lCbeLoCbe8-10
Right cerebellar vermal lobules VIII-X	rCbeLoCbe8-10
Left brainstem	lBst
Right brainstem	rBst

The 26 ROIs were taken from the Neuromorphometrics atlas, which is shipped with the CAT12 toolbox. Anatomical labelling of the ROIs and ROI abbreviations are adopted from the CAT12 toolbox (terms in brackets have been added for intuitive understanding).

Supplementary results table

Supplementary Table 2. Relationship of motor symptoms severity with clinical characteristics.

		Correlation statistics	Correlation statistics
		**Dyskinesia (AIMS)**	**Parkinsonism (SAS)**
Age at disease manifestation (years)	25.2 ± 8.5	$r_s = 0.300$; $p = 0.089$	$r_s = 0.198$; $p = 0.269$
Disease duration (years)	13.5 ± 10.8	*$r_s = 0.712$; $p < 0.001$*	$r_s = 0.253$; $p = 0.115$
Times of being inpatient	6.8 ± 7.6	*$r_s = 0.538$; $p = 0.002$*	$r_s = 0.284$; $p = 0.121$
Concomittant antipsychotic dose*	662.5 ± 848.3	$r_s = -0.088$; $p = 0.615$	$r_s = 0.003$; $p = 0.986$
Psychopathology (BPRS)	40.1 ± 11.0	$r_s = -0.138$; $p = 0.431$	$r_s = -0.103$; $p = 0.557$

Spearman rank correlations showed that severity of dyskinesia and parkinsonism is not linked to early disease manifestation, high concomitant antipsychotic dose, or severity of psychopathological symptoms. Dyskinesia severity was positively associated with disease duration and times of being inpatient, i.e., indicators of chronification of disease. Values are reported as mean $\pm$ standard deviation and correlation statistics as both the Spearman rank correlation coefficient r_s and the 2-sided significance value p. Significant correlation are presented in italics.
AIMS = Abnormal Involuntary Movement Scale[1]; BPRS = Brief Psychiatric Rating Scale[2]; SAS = Simpson Angus Scale[3]
*Doses equivalents to 100 mg chlorpromazine were calculated according to Leucht *et al.*[4].

References

1. Guy W, Ban TA, Wilson WH. An international survey of tardive dyskinesia. *Prog Neuropsychopharmacol Biol Psychiatry*. 1985;9(4):401-5. doi:10.1016/0278-5846(85)90193-9
2. Overall JE, Gorham DR. The Brief Psychiatric Rating Scale. *Psychological Reports*. 1962;10(3):799-812. doi:10.2466/pr0.1962.10.3.799
3. Simpson GM, Angus JW. A rating scale for extrapyramidal side effects. *Acta Psychiatr Scand Suppl*. 1970;212:11-9. doi:10.1111/j.1600-0447.1970.tb02066.x
4. Leucht S, Samara M, Heres S, Davis JM. Dose Equivalents for Antipsychotic Drugs: The DDD Method. *Schizophr Bull*. Jul 2016;42 Suppl 1(Suppl 1):S90-4. doi:10.1093/schbul/sbv167
